# Quality Evaluation of Commercial Products Marketed as *Thymus vulgaris* L. Essential Oil According to European Pharmacopoeia 10.0 Criteria

**DOI:** 10.3390/molecules31193513

**Published:** 2026-10-02

**Authors:** Nehir Kavi, İrem Şevval Bayri, Tuğba Buse Şentürk, Timur Hakan Barak, Engin Celep

**Affiliations:** 1 Department of Pharmacognosy, Faculty of Pharmacy, Acibadem Mehmet Ali Aydinlar University, Istanbul 34752, Türkiye; nehir.kavi@acibadem.edu.tr (N.K.); tugba.avci@acibadem.edu.tr (T.B.Ş.); engin.celep@acibadem.edu.tr (E.C.); 2 Institute of Graduate Studies in Health Sciences, Istanbul University, Istanbul 34126, Türkiye; 3 Faculty of Pharmacy, Acibadem Mehmet Ali Aydinlar University, Istanbul 34752, Türkiye; irem.bayri@live.acibadem.edu.tr

**Keywords:** *Thymus vulgaris* L., essential oil, European Pharmacopoeia, GC-MS, quality control

## Abstract

Essential oils are mixtures of complex secondary metabolites with variable chemical compositions that determine their therapeutic effects. Thyme oil, obtained from the fresh or dried leaves and flowers of *Thymus* spp. through steam distillation, is widely used in food, pharmacy, and perfumery–cosmetics due to its antimicrobial, antitussive, and antioxidant properties. Ensuring the quality of essential oils is important for public health, as globally encountered deviations from the established standards pose a risk to consumers. This study evaluated the quality of 12 different *Thymus vulgaris* L. essential oil (TVEO) samples from pharmacies and various sources on the Turkish market, focusing on their adherence to the European Pharmacopoeia (EP) 10.0. Following primary assessments of appearance, solubility, relative density and refractive index; high-performance thin-layer chromatography (HPTLC) and gas chromatography–mass spectrometry (GC-MS) analyses were performed for each TVEO sample to profile their phytochemical composition. Results revealed that none of the samples met all the criteria specified in EP 10.0. The mean proportion of EP criteria fulfilled per sample is 46.9% for pharmacy-sourced samples and 41.9% for non-pharmacy-sourced samples. The findings of this study suggest that future quality control analysis for TVEO samples on the Turkish market should integrate comprehensive chemical profiling with advanced analytical strategies.

## 1. Introduction

Essential oils are widely used natural products that have been integrated into daily life across the world. High amounts of essential oils are produced to meet the needs of the cosmetics, food flavouring, and health industries [1]. Historically, *Thymus vulgaris* L. (Lamiaceae) has been used in respiratory diseases such as bronchitis, asthma and cough. The pharmacological activity of the essential oil is primarily attributed to its main constituent, thymol, which exhibits antifungal, antibacterial, and antioxidant properties, as well as its isomer, carvacrol, which possesses reported acetylcholinesterase inhibitory, antimicrobial, anti-inflammatory, and antithrombotic actions [2,3]. Therapeutic applications include consumption as herbal teas or syrups, topical application as ointments, and use through steam inhalation [4]. The origin of the name “*Thymus*” is believed to come either from the Greek word “thyo” (perfume) or from another Greek word “thymos” (courage, strength). Dioscorides mentions “thymo” in his works about medicinal plants. The genus *Thymus* is widely distributed throughout the Mediterranean basin [5]. In Türkiye, the total number of *Thymus* species has increased to 39 with the addition of a new member called “*T. baseri*” [6,7]. Locally known as “kekik”, dried parts of *Thymus* spp. are commonly used to make herbal teas in traditional medicine or as food seasoning. However, there are four more genera that are referred to locally as “kekik”, *Origanum*, *Thymbra*, *Coridothymus* and *Satureja*, due to the presence of major chemical components in the essential oils, responsible for the distinct smell associated with thyme [8]. Thyme oil has been used for centuries, though its extraction was officially documented in the 1930s. In 2020, global production hit 191.45 thousand metric tons, contributed by North and Latin America, Europe, the Middle East, Asia-Pacific, and Africa. The global market is expected to grow by 7.8% by 2027 [4]. However, the growing demand in the market raises questions about the authenticity of the commercially available products.

According to the European Pharmacopoeia (EP), TVEO should be obtained by performing steam distillation from the fresh flowering aerial parts of *Thymus vulgaris* L., *T. zygis* L., or a mixture of these species [9]. Pharmacopoeia provides standard values for both natural-origin and synthetic substances used in drug manufacturing to ensure quality. Türkiye is associated with the EP to validate compliance with international standards. On the Turkish market, various natural products claim to contain pure essential oils on their label. Considering that essential oil composition is highly sensitive to variables including plant origin, harvesting conditions, extraction techniques, and storage, the assessment of the quality of such products is crucial. Furthermore, without sufficient quality assessment, several concerns, including mislabelling, adulteration, contamination, oxidation, or degradation, may occur. Failure to adhere to international standards raises potential safety and quality concerns, considering these products may be utilised for therapeutic purposes. Essential oils that fall short of meeting EP requirements could fail to perform the anticipated biological activity and may cause allergic reactions or other unfavourable side effects. Hence, standardisation in accordance with EP criteria has inevitably become a necessity. To our knowledge, no particular research has been conducted on TVEO focused primarily on EP standards assessing the quality of such products available on the market, despite the wide range of analyses in the literature.

In this study, TVEO samples from 12 commercial brands all claiming absolute purity on their labels were analysed according to the EP 10th edition, which was the applicable version at the time of the analysis when the samples were collected. In order to assess quality variations among distribution channels, two samples were purchased from pharmacies, while the remaining 10 were obtained from herbal shops or online retailers. Quality evaluation in the current study involved high-performance thin-layer chromatography (HPTLC), appearance and solubility tests for visual assessment, alongside relative density and refractive index measurements. Furthermore, gas chromatography–mass spectrometry (GC-MS) analysis was performed to characterise the phytochemical profiles of these essential oils by comparing the percentage levels of ten constituents present in the EP monograph titled “thyme oil, thymol type”. Therefore, this study aimed to complement the limited literature data and provide insights into potential quality and safety challenges within the essential oil production industry.

## 2. Results

### 2.1. Appearance

European Pharmacopoeia 10.0 criteria specify that TVEO should be a clear, yellow or very dark reddish-brown, mobile liquid. All samples complied with the EP 10.0 criteria (Table 1).

### 2.2. Solubility

#### 2.2.1. Solubility in Anhydrous Ethanol

TVEO should be miscible with anhydrous ethanol according to the European Pharmacopoeia. All samples were found to be compatible with the EP 10.0 criteria (Table 1).

#### 2.2.2. Solubility in Light Petroleum

TVEO should be miscible with light petroleum according to the European Pharmacopoeia. All samples were found to be compatible with the EP 10.0 criteria (Table 1).

### 2.3. Relative Density, Refractive Index

The relative density value for TVEO should be between 0.915 and 0.935 according to the European Pharmacopoeia 10.0 criteria. Only samples P1, A2, A4, A5, A10 were in accordance with the criteria (Table 1).

The refractive index value for TVEO should be between 1.490 and 1.505 according to the European Pharmacopoeia 10.0 criteria. Only samples P1, A2, A4, A5, A7, and A10 were in accordance with the criteria (Table 1).

### 2.4. Chromatographic Profile

#### 2.4.1. High-Performance Thin-Layer Chromatography (HPTLC)

**Figure 1:**
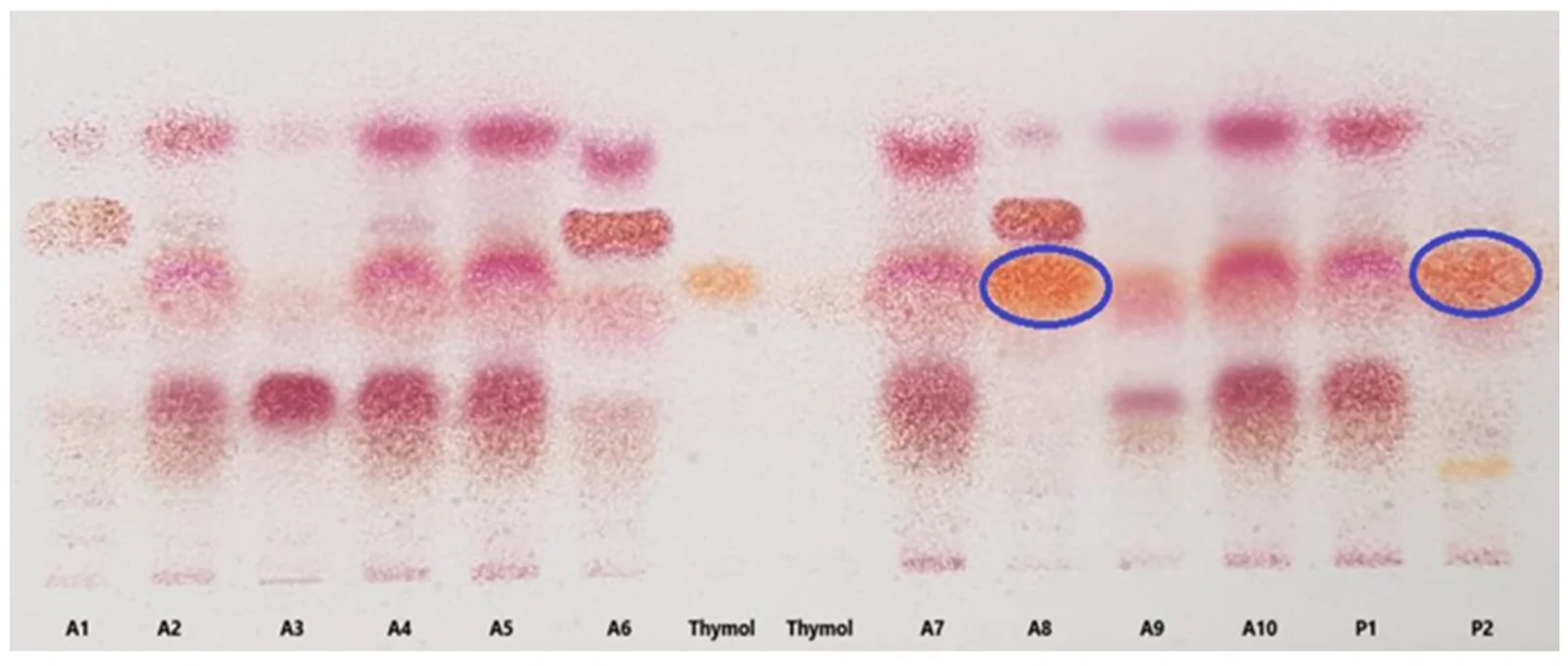
The results of HPTLC analysis for all samples. Thymol: Thymol standard. Spots marked with a blue circle are thymol.

Following EP, thymol should appear in an intense orange-brown region (top) and carvacrol in a pale orange-grey region may be present. Based on the results seen on the HPTLC plate, thymol was observed in samples A8 and P2 (see Figure 1).

#### 2.4.2. Gas Chromatography–Mass Spectrometry (GC-MS)

**Figure 2:**
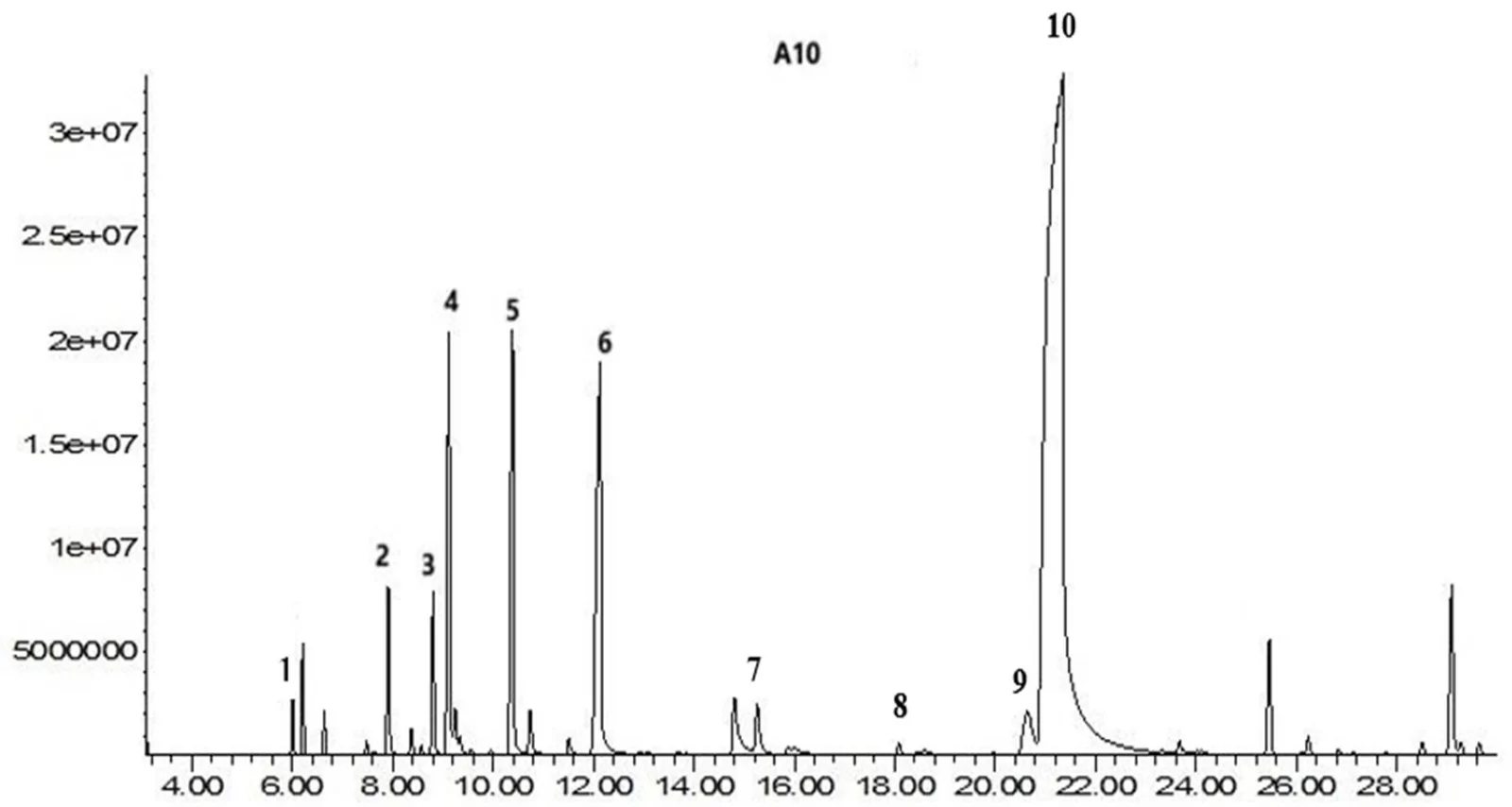
GC-MS chromatogram of A10-coded sample. 1: α-thujene, 2: β-myrcene, 3: α-terpinene, 4: p-cymene, 5: γ-terpinene, 6: linalool, 7: terpinene-4-ol, 8: carvacrol methyl ether, 9: thymol, 10: carvacrol.

The findings of GC-MS analysis highlighting the phytochemical profile of 12 samples are presented in Table 2. Thirty distinct chemical components were revealed. TVEO chemical profile was resolved to a minimum of 96.3% and a maximum of 100.0%. Figure 2 illustrates 10 phytochemical components included in the EP with a typical chromatogram example of the sample coded as A10. In order to validate the findings, a reference/standard mixture was used. This reference mixture contained selected chemical components that were commonly found in essential oils. Table 3 shows the compatibility of the GC-MS analyses to EP.

## 3. Discussion

Essential oils are natural mixtures of various chemical constituents that are used in a wide range of industries worldwide and are now an essential part of daily life. The growing demand for essential oils has led to several problems such as authentication failure and adulteration. Intentional adulteration is generally motivated by economic gain, whereas unintentional or inadvertent adulteration frequently results from the lack of knowledge of proper equipment use or accurate plant identification [10]. It is widely recognised that the source of the species has a significant impact on the amount and the quality of herbal products. The pharmacological effects and active constituents of a given herbal drug from different producing areas, or distinct herbal products within the same species, may change significantly. In order to maintain the standardisation and quality of products from various species and geographical areas, it is crucial to authenticate the plant material by correctly identifying the plant species, tracing its origin, and determining the harvest time as well as its growth stage [11]. The lack of reliable data concerning the authenticity and quality of commercial essential oil products is particularly concerning, given their importance in both medicine and cosmetics. The findings of similar studies confirm the considerable discrepancy between the product composition and label claims. Therefore, the main objective was to highlight quality-related concerns in essential oil-containing products and to raise awareness of the potential risks these issues may pose to consumer safety.

In the case of herbal products, quality control of essential oils relies on EP monographs, which set international standards by defining physical properties alongside expected phytochemical profiles and concentration limits [9]. It is noticeable that the literature contains only a limited number of studies about commercially available essential oil product compatibility with the EP. In a previous study, 15 distinct rosemary essential oil samples that were gathered from various Turkish retailers underwent quality control analysis according to the EP. None of the samples completely met the requirements according to the findings of the study. However, compared to the samples that were bought from other sources, pharmacy-oriented samples showed more acceptable results [12]. Several studies support the obtained data. Some eucalyptus, pine turpentine, and clove essential oil samples that were collected from various commercial sources and their compliance with EP requirements were previously assessed. In a comparable manner, no sample was shown to completely fulfill the requirements [13,14,15]. In a similar study clove bud oil, neroli oil, tea tree oil, and cinnamon oil samples were evaluated according to the Turkish Pharmacopoeia (2017). Among 25 essential oils in total, only two of them were compatible with the pharmacopoeia [16].

Although the phrase “pure essential oils” is widely used as a marketing strategy for a variety of products, quality discrepancies have been increasingly reported, mainly due to the high costs associated with high-quality essential oil-containing products compared to synthetic alternatives. Twelve samples of TVEO from various brands were acquired for the present study. Each sample was checked to validate the “100% pure essential oil” label statement prior to the analysis. Firstly, all samples were analysed for their appearance and solubility characteristics and were found to be compatible with the EP. However, more than half of the samples failed to comply with the specified criteria for refractive index and relative density (Table 1). Both parameters are widely used as indicators of essential oil purity and quality. Delgado Ospina et al. [17] reported that the refractive index of an essential oil is primarily determined by its major constituent, while also being influenced by synergistic interactions with the other components. Thus, the discrepancy of such results has given the first signals of an imbalance in the chemical composition. In the current study P2- and A8-coded samples were found to be the only samples containing the required amounts of thymol (Table 3), while their low refractive index values can be interpreted using the combined refractive indices of the second- and third-most abundant components in the relevant essential oil samples. Later on, the evaluation continued with high-performance thin-layer chromatography (HPTLC) and gas chromatography–mass spectrometry (GC-MS) analyses. Thyme oil is predominantly composed of monoterpene groups, with thymol (a phenolic monoterpene) being the major constituent (30–55%). Thymol is accompanied by several biosynthetically related monoterpenes, especially its isomer carvacrol (1–5%). Functioning as biogenetic precursors, p-cymene (15–20%) and γ-terpinene (5–10%) are essential components that lead to the synthesis of both thymol and carvacrol [18]. The apparent discrepancy in the HPTLC profile may be attributed to the close structural similarity between thymol and carvacrol. As positional isomers, these compounds may exhibit similar chromatographic responses (Figure 1). No carvacrol presence was detected in the P2- and A8-coded samples in the results of GC-MS analysis (Table 2). Consequently, these samples exhibited the intense orange-brown zone characteristics of thymol on the HPTLC result plate, as specified by the EP. Additionally, among all the samples analysed, only the samples coded P2 and A8 contained thymol at the required level in the GC-MS results (Table 3). However, the other samples displayed a mixture of colours in the specific R_f_ region corresponding to thymol. Given the difficulty of chromatographic separation of thymol and carvacrol, there is a high chance that co-migration or overlap of these two isomers likely occurred in the remaining samples, explaining the observed colour variation [19].

Previously, 85% of the *Thymus* taxa of Türkiye were investigated for their essential oil composition. Almost half of those were revealed to be rich in essential oils, bearing more than 1% essential oil content. A previous study identified thymol as the major constituent in 24 *Thymus* taxa and carvacrol in 11 taxa [20]. *Thymus vulgaris* is known to have chemical polymorphism with several chemotypes, including thymol type, carvacrol type, linalool type, geraniol type, sabinene hydrate type, and more [21]. EP 10.0 outlines only the monograph for “thyme oil, thymol type” while other chemotypes are excluded. Since no information on chemotype was provided on the product labels, all samples were evaluated according to the specifications of the existing monograph. However, different chemotypes may occur in close geographical proximity and may even coexist within the same plant population [22]. It is known that the reason for their odour variety is attributed to differences in major components. The thyme-scented chemotype was characterised by thymol (53%) and *p*-cymene (18%) as its major constituents, whereas the rose-scented chemotype was dominated by geraniol (69%) and geranyl acetate (16%) (Başer, 2002 [20]). The current study indicated that nine different samples (P1, A1, A2, A4, A5, A6, A7, A9, and A10) possess carvacrol as the major component instead of thymol. In addition to that, the A3-coded sample showed linalool as the major constituent, supporting the hypothesis of Başer et al. [22]. The GC–MS findings may also be interpreted in light of factors other than chemotype variation. Both intrinsic factors, such as seasonal and ontogenetic changes, and extrinsic influences, like soil composition, climate, and light exposure, have a direct influence on leading to these varieties [18]. For instance, an essential oil sample obtained from *Thymus vulgaris* was shown to have the highest percentage of *p*-cymene and the lowest amount of thymol in May. Later, these levels were found to remain constant during the vegetation period. Additionally, it was revealed that June and early July had relatively lower α-terpinene concentrations. However, as the percentage of thymol began to decrease in late June and early August, the concentration of α-terpinene demonstrated a significant increase, indicating that the content of essential oil is greatly influenced by the time of harvest. Therefore, detecting the appropriate stage of development for plant harvesting is of crucial importance for the maximisation of the quantity and quality of thyme essential oil. In addition, differences in the method of extraction have major impacts on the composition of thyme oil. Studies revealed that the number of key components might be diminished when plant material of *T. vulgaris* is dried using hot air or microwave ovens. Microwave treatment of the plant material resulted in a considerable decrease in the relative thymol content of the essential oil [4]. However, beyond environmental and chemotypic variations, the atypical profile of the A8-coded sample—characterised by the absence of minor constituents while detecting 10 compounds that correspond to 100% of the oil—suggests the possibility of a reconstituted or adulterated product. This finding supports the rest of the results and indicates that the chemical profile of the A8-coded sample deviates considerably from that of an authentic distillate.

Another possibility for the high carvacrol content in most of the samples (P1, A1, A2, A4, A5, A6, A7, A9, A10) can be attributed to the commercial use of *Origanum* taxa in Türkiye instead of non-native *T. vulgaris*, since carvacrol is considered to be the major component in *Origanum* species. While the number of *Origanum* taxa native to the Mediterranean regions exceeds 45, Türkiye alone hosts 21 of them [23]. As stated earlier, more than 50 taxa are locally known as thyme in Türkiye, but many of them belong to several other genera, including *Origanum* [24]. Thyme essential oil export has been on the rise in recent years. In 2019, an export volume of 30 tons of thyme essential oil yielded $3 million in returns [25]. In light of this information, the processing industry in Türkiye is prone to invest in the cultivation of *Origanum* species for the commercial production of thyme essential oil instead of *Thymus vulgaris*. Considering that different genera are all colloquially referred to as “thyme/kekik”, this ambiguity is highly expected if regulatory authorities fail to address the nomenclature. However, this potential botanical substitution remains a plausible hypothesis, as GC-MS compositional analysis alone is insufficient to definitively distinguish between chemotype variations, botanical substitution, or intentional adulteration.

Products purchased from pharmacies showed a higher mean criteria fulfilment rate with the EP than those obtained from alternative sources (46.9% and 41.9%, respectively). These rates were first determined for each sample by dividing the number of passed tests by the total number of 16 tests (Table 1 and Table 3). The result is then expressed as a percentage. For each sample, this process is repeated. The average of these percentages was then calculated for both P-coded and A-coded samples independently. While samples must strictly adhere to EP specifications to achieve full compliance, none of the tested samples met all criteria; therefore, the mean proportion of fulfilled criteria was determined. Although these values are insufficient to demonstrate clear superiority, these figures accurately reflect the obtained data. Essential oils that are freely accessible in unregulated markets create a risk of introducing lower-quality products to the market. Adherence to pharmacopoeia standards improves the reliability of the products and protects consumer health worldwide. By defining acceptable limits for the compositional differences in essential oils caused by various factors, pharmacopoeia monographs establish clear quality specifications and provide a reliable framework for their consistent and appropriate therapeutic use. While the findings of this study were initially expected to shed light on possible adulteration, they revealed other challenges that can occur during the production of natural products, such as chemotype diversity, botanical misidentification, or differences in cultivation, harvesting, and extraction. In this context, the findings emphasise that ensuring product quality and chemical composition consistency requires rigorous authentication standards and comprehensive quality-control procedures throughout the entire supply chain.

## 4. Materials and Methods

### 4.1. Sample Collection

Twelve samples of different brands were collected from different sources in Türkiye, two of the samples were collected from pharmacies, and 10 of the samples were collected from various sources, including herbalists and web-based markets other than pharmacies. During the collection process, care was taken to verify that the samples were claimed to be 100% pure TVEO, obtained from *Thymus vulgaris*, and that their records had been verified in the database of Ministry of Health of the Republic of Türkiye. The samples are coded according to the purchase channel. Products purchased from pharmacies are coded with “P”, while products purchased from other channels are coded with “A”. Samples were stored at room temperature in tightly closed containers and away from sunlight until the products were tested according to the EP. All standards were sourced from Sigma-Aldrich (St. Louis, MA, USA).

### 4.2. Appearance Test

All analytical procedures were conducted in accordance with the EP, under slight modifications from the original protocol. For the appearance, equivalent volumes of the 12 samples were transferred into individually coded test tubes. Visual examinations were carried out to determine whether the colour and clarity of each sample meet EP standards.

### 4.3. Solubility Test

#### 4.3.1. Solubility in Anhydrous Ethanol

A test tube was filled with 5 mL of anhydrous ethanol solution, and then 1 mL of the sample was added. The mixture of anhydrous ethanol and sample solution was stirred thoroughly to assess complete miscibility. This assay was performed individually for each sample.

#### 4.3.2. Solubility in Light Petroleum

A test tube was filled with 5 mL of light petroleum solution, and then 1 mL of the sample was added. The mixture of light petroleum and sample solution was stirred thoroughly to assess complete miscibility. This assay was performed individually for each sample.

### 4.4. Relative Density, Refractive Index

Relative density assays were determined gravimetrically in triplicate at a controlled temperature of 20.0 °C following the EP principles. A calibrated analytical balance and a calibrated micropipette were used. The system was calibrated with distilled water prior to analysis. The assays were conducted by evaluating the weight of 1 mL of essential oil and comparing it against the density of distilled water at 20.0 °C. The determination was made by weighing; therefore, the buoyancy of air is disregarded, as the EP states. Refractive index measurements were performed with an Anton Paar (Graz, Austria)-Abbemat 3100 refractometer. Once the device was equilibrated to 20 °C, a drop of the TVEO sample was dropped onto the sample compartment with the help of a Pasteur pipette, and the measurement was initiated. The recorded data were documented, and the mean values were calculated. Both assays were performed in triplicate.

### 4.5. High-Performance Thin-Layer Chromatography (HPTLC)

HPTLC was performed in accordance with the instructions present in the “Thyme Oil, Thymol Type” monograph under the “Thin Layer Chromatography” title, with minor adaptations to use CAMAG HPTLC Automatic TLC Sampler 4 ATS 4 and CAMAG HPTLC Automatic Developing Chamber ADC 2 (Muttenz, Switzerland) [12]. Sample solutions were prepared by dissolving 0.2 mL of each sample in methylene chloride R and diluting to 10 mL with the same solvent. The reference solution was prepared by dissolving 5 mg of thymol R in methylene chloride R and diluting to 10 mL with the same solvent. Silica gel F254 plates were used as the stationary phase, and methylene chloride R was used as the mobile phase. The sample and reference solutions were applied as 10 mm bands, with an application volume of 10 µL. Before development, the ADC 2 chamber was saturated and pre-conditioned with MgCl_2_ at 33% relative humidity for 10 min. The plates were then developed over a distance of 12 cm. After development, the plates were air-dried, sprayed with anisaldehyde solution R, and heated at 100–105 °C for 5 min. The chromatograms were examined under daylight.

### 4.6. Gas Chromatography–Mass Spectrometry (GC-MS)

Gas chromatography–mass spectrometry analyses were conducted according to the Üstüner et al. [26] method. Volumes of 20 µL of TVEO samples were diluted in 180 µL of n-hexane (GV grade, Sigma-Aldrich). A volume of 1 µL from each sample was injected with a split mode of 1:50 (Agilent Technologies, Santa Clara, CA, USA). An HP-5MS capillary column (Agilent Technologies, Santa Clara, CA, USA) (5% diphenyl, 95% dimethyl polysiloxane; 30 m × 0.25 mm, 0.25 µm film thickness) was used. The column oven was held at 60 °C for 1 min, increased to 246 °C at 3 °C/min, and sustained at 246 °C for an additional 30 min. Helium was used as the carrier gas (0.9 mL/min). Mass spectrometry was used as the detector. These conditions were maintained for both the standard mixture and all essential oil runs. Volatile components were first characterised by cross-matching their mass spectra with NIST17 and Wiley7 digital libraries, and then by comparing their RRI (Relative retention index) that is calculated by previously injected C8–C40 n-alkane series data with the literature. Relative percentages were calculated based on the integrated MS chromatogram peak areas.

## 5. Conclusions

Essential oils serve as valuable plant-derived products with prophylactic and therapeutic actions across many industries, particularly in pharmacy. Consequently, adherence to international criteria is important to ensure their place on the market as high-quality products. The widespread commercial availability of essential oils on various non-pharmacy platforms highlights the gap in regulatory oversight, pointing out the necessity for rigorous quality control. Such safety and quality concerns associated with consumer-ready natural products remain undesirable, demanding strict compatibility with officially recognised pharmacopoeias. This study represents efforts in the literature regarding the quality of commercial *Thymus vulgaris* essential oil (TVEO) samples on the Turkish market; up-to-date quality analyses were performed based on the EP 10.0. Notably, none of the 12 samples displayed absolute compliance with all monograph specifications. The lowest compatibility was observed with A3- and A9-coded samples, fulfilling merely four out of 16 criteria, whereas sample A10 exhibited relatively higher compliance by fulfilling 11 out of 16 criteria. Descriptively, the mean proportion of fulfilled EP criteria per sample is 49.6% for pharmacy-sourced TVEO samples compared to 41.9% for those purchased from non-pharmacy sources. However, the obtained data should be interpreted with caution given the unbalanced sample sizes. Yet, the overall fulfilment remains inadequate across both groups. The chemical fluctuations observed herein display the complex challenges embedded in natural product manufacturing. A primary finding of this study is the pronounced chemical variability among the evaluated commercial products, which can be categorised into three distinct chemical profiles: carvacrol-rich (the majority of the samples), thymol-rich (P2- and A8-coded samples), and linalool-rich (A3-coded sample). This is particularly worth mentioning since the EP explicitly requires a thymol-dominated profile for thyme oil. Therefore, carvacrol-dominant samples emphasise a significant compositional deviation. These deviations may arise from the utilisation of a distinct *Thymus vulgaris* chemotype rather than the specific one defined by the pharmacopoeia, or from the intentional/unintentional substitution with alternative plant species that emit a similar scent. Additionally, environmental cultivation factors, harvesting schedules, and technical extraction variations during the distillation process need to be underlined, as it alters the final chemical composition. Mentioned variability compromises the reliable authentication of genuine *Thymus* species and reveals that commercial labelling often misrepresents the true chemical constituents. However, this study has several limitations, including the total sample number, the absence of direct botanical authentication, the uneven distribution between sample sources, and GC-MS remains limited in definitively distinguishing chemotype variations from botanical substitution or adulteration. Due to natural biological shifts, processing variables, and fraudulent practices are challenging to predict; future quality control regulations for TVEO in the Turkish marketplace must be broadened by combining systemic chemical profiling with chemometric analysis.

## Figures and Tables

**Table 1:** General results table.

TESTS	Reference Interval	P1	P2	A1	A2	A3	A4	A5	A6	A7	A8	A9	A10
Appearance	Clear, yellow or very dark reddish-brown liquid	+	+	+	+	+	+	+	+	+	+	+	+
Solubility with ethanol	Miscible with anhydrous ethanol	+	+	+	+	+	+	+	+	+	+	+	+
Solubility with light petroleum	Miscible with light petroleum	+	+	+	+	+	+	+	+	+	+	+	+
Relative Density	0.915 to 0.935	0.925 ± 0.000	0.890 ± 0.000	0.888 ± 0.000	0.935 ± 0.000	0.797 ± 0.001	0.923 ± 0.000	0.929 ± 0.000	0.882 ± 0.000	0.890 ± 0.000	0.891 ± 0.000	0.960 ± 0.001	0.918 ± 0.000
Refractive Index	1.490 to 1.505	1.504	1.452	1.475	1.502	1.392	1.500	1.495	1.482	1.505	1.479	1.463	1.501
HPTLC		−	+	−	−	−	−	−	−	−	+	−	−

+: indicates pass; −: indicates fail.

**Table 2:** Phytochemical profile of TVEO samples in percentage.

Compound	RT	RI^exp^	RI^lit^	RI^std^	P1	P2	A1	A2	A3	A4	A5	A6	A7	A8	A9	A10
α-Thujene	6.10	926	927						0.62			1.54		0.3	0.14	0.50
α-Pinene	6.26	931	933	928	0.57	0.26	0.56	0.44	0.50	0.30	0.6		0.39	0.57	0.14	1.03
Camphene	6.63	948	949	948	0.25		0.25	0.18		0.13	0.35	0.72	0.14			0.43
β-Pinene	7.48	978	978			1.68								3.42		
β-Myrcene	7.90	989	993	986	1.15	0.24	0.97	0.93	0.67	0.65	0.72	0.29	0.68	0.50	0.27	1.80
α-Phellandrene	8.37	1002	1007		0.23			0.22		0.15	0.16	0.29	0.16			0.29
α-Terpinene	8.65	1010	1017	1013	1.24	0.14		0.97	0.52	0.78	0.83	1.63	0.74	0.23	0.23	1.87
*p*-Cymene	9.35	1028	1031		4.92	22.1	4.65	4.23	3.28	3.05	5.79	5.68	4.52	23	2.10	
Limonene	9.20	1024	1030	1024	0.39	1.27		0.41		0.3	0.55	0.47	0.34			
1,8-Cineol	9.30	1027	1033	1032							1.51					
γ-Terpinene	10.4	1056	1059		3.13	27.0	3.85	2.98	1.25	2.48	1.83	6.33	1.92	23.5	1.09	5.98
α-Terpinolene	11.5	1085	1089	1091				0.29	0.62	0.26	0.3			1.19	3.1	0.28
Linalool	12.1	1101	1101	1098	9.82		4.86	8.11	57.9	12.6	8.18	4.47	7.14		10.6	8.73
β-Thujone	12.7	1115	1117								0.21					
*endo*-Borneol	14.8	1165	1168	1164	1.67		1.08	2.31		1.99	2.03	1.25	1.57	0.36	1.54	1.62
Terpinene-4-ol	15.2	1175	1179	1174	1.51		0.84	1.73		1.22	1.40	1.30	1.41		0.88	1.08
α-Terpineol	15.9	1190	1195	1189				0.37	0.36				0.24		0.22	
Carvacrol Methyl ether	18.1	1241	1245		1.17			0.32		0.32	0.20	0.43	0.18			0.19
Thymol	20.8	1303	1305	1304	2.05	45.9	2.47	3.43	31.8	1.70	2.23	1.28	2.42	46.9	1.45	1.81
Carvacrol	21.3	1316	1317	1315	65.2		74.6	64.1		68.2	61.9	63.7	70.8		62.9	59.9
Carvacryl acetate	23.7	1372	1371		0.28			0.6		0.19	0.22		0.54			0.23
β-Caryophyllene	25.4	1414	1421	1412	1.48		1.47	1.36	1.7	1.31	1.58	1.96	0.99		0.68	1.77
Aromadendrene	26.1	1431	1439		0.25			0.19		0.19	0.3					0.28
Humulene	27.2	1459	1458							2.86	0.23					
β-Bisabolene	29.1	1505	1506	1508	3.10		2.00	3.96			3.76	4.35	3.66		1.93	4.6
γ-Cadinene	29.3	1509	1511					0.34			0.36	0.36	0.31			0.23
Spathulenol	31.8	1575	1577		0.18			0.23			0.31		0.23			0.22
Caryophyllene oxide	31.9	1579	1583	1579	0.55		0.44	0.5		0.45	0.86		0.32		0.54	0.54
Viridiflorol	32.3	1589	1587								0.3		0.46			
τ-Cadinol	34.2	1638	1640		0.38		0.44		0.29	0.53		0.23				0.4
			Total %		99.5	98.5	98.8	98.2	99.5	99.7	96.8	96.3	99.2	100	99.8	99.3

RT: Retention time, RI^exp^: Retention index of the experiment, RI^lit^: Retention index in the literature, RI^std^: Retention index of the reference/standard mixture.

**Table 3:** Results of the GC-MS analyses.

Components	Reference Interval (%)	P1	P2	A1	A2	A3	A4	A5	A6	A7	A8	A9	A10
α-Thujene	0.2–1.5					0.62			1.54		0.3	0.14	0.50
β-Myrcene	1.0–3.0	1.15	0.24	0.97	0.93	0.67	0.65	0.72	0.29	0.68	0.50	0.27	1.80
α-Terpinene	0.9–2.6	1.24	0.14		0.97	0.52	0.78	0.83	1.63	0.74	0.23	0.23	1.87
*p*-Cymene	14.0–28.0	4.92	22.1	4.65	4.23	3.28	3.05	5.79	5.68	4.52	23.0	2.10	
γ-Terpinene	4.0–12.0	3.13	27.0	3.85	2.98	1.25	2.48	1.83	6.33	1.92	23.5	1.09	5.98
Linalool	1.5–6.5	9.82		4.86	8.11	57.9	12.6	8.18	4.47	7.14		10.6	8.73
Terpinene-4-ol	0.1–2.5	1.51		0.84	1.73		1.22	1.40	1.30	1.41		0.88	1.08
Carvacrol methyl ether	0.05–1.5	1.17			0.32		0.32	0.20	0.43	0.18			0.19
Thymol	37.0–55.0	2.05	45.9	2.47	3.43	31.8	1.70	2.23	1.28	2.42	46.9	1.45	1.81
Carvacrol	0.5–5.5	65.2		74.6	64.1		68.2	61.9	63.7	70.8		62.9	59.9

## Data Availability

The raw data supporting the conclusions of this article will be made available by the authors on request.

## Abbreviations

TVEO	*Thymus vulgaris* essential oil
EP	European Pharmacopoeia
HPTLC	High-performance thin-layer chromatography
GC-MS	Gas chromatography–mass spectrometry
